# Effects of regular, glulisine, and aspart insulin on vascular endothelial growth factor and angiotensinogen expression in hyperglycemic retinal pigment epithelial (RPE) and human retinal endothelial cells (HRECs)

**DOI:** 10.3389/fopht.2025.1570232

**Published:** 2025-05-29

**Authors:** Fatemeh Sanie-Jahromi, Abtin Khosravi, Hooman Hadianfard, M. Hossein Nowroozzadeh

**Affiliations:** Poostchi Ophthalmology Research Center, Department of Ophthalmology, School of Medicine, Shiraz University of Medical Sciences, Shiraz, Iran

**Keywords:** angiotensinogen, aspart, diabetic retinopathy, glulisine, human retinal endothelial cells, insulin, retinal pigment epithelium, vascular endothelial growth factor

## Abstract

**Introduction:**

Diabetic retinopathy (DR) is a leading cause of vision loss and is primarily driven by chronic hyperglycemia, which induces retinal vascular damage through mechanisms involving vascular endothelial growth factor (VEGF) and the renin-angiotensin system (RAS). This study investigated the effects of hyperglycemia and different insulin formulations—regular, glulisine, and aspart—on VEGF-A and angiotensinogen (AGT) gene expression in two human retinal cell types: retinal pigment epithelial (RPE) cells and human retinal microvascular endothelial cells (HRECs).

**Methods:**

Cells were cultured from donor tissue and exposed to physiologic and hyperglycemic glucose concentrations, with or without insulin treatment. Gene expression levels were quantified using real-time PCR.

**Results:**

Hyperglycemia significantly upregulated VEGF-A and AGT in both RPE and HREC cells (e.g., VEGF-A in RPE: 2.62-fold, *P* = 0.001; AGT in RPE: 3.32-fold, *P* = 0.093), supporting a role for both osmotic and glucose-specific pathways. Among insulin treatments, regular insulin significantly reduced VEGF-A expression in both RPE (0.72-fold, *P* = 0.033) and HRECs (0.57-fold, *P* = 0.009). In contrast, aspart and glulisine had modest effects on VEGF-A in HRECs (0.82-fold each; *P* = 0.035 and *P* = 0.060, respectively) and no significant impact in RPE cells. Regarding AGT, aspart insulin showed the most consistent suppressive effect, reducing expression in both RPE (0.15-fold, *P* < 0.001) and HRECs (0.22-fold, *P* = 0.004). Glulisine significantly increased AGT in RPE (1.56-fold, *P* = 0.009) but reduced it in HRECs (0.58-fold, *P* = 0.074). Regular insulin showed no effect on AGT in RPE (*P* = 0.680) and a non-significant increase in HRECs (1.36-fold, *P* = 0.097).

**Discussion:**

These findings highlight the differential biological effects of insulin analogues and suggest that aspart insulin, in particular, may offer therapeutic benefits beyond glycemic control by modulating both VEGF-A and RAS-related pathways. Tailored insulin therapies could represent innovative strategies for managing or slowing the progression of diabetic retinopathy.

## Introduction

Diabetes is characterized by hyperglycemia resulting from an absolute or relative deficiency in insulin production or action (1). Early microvascular complications of diabetes commonly affect organs such as the eyes, kidneys, and nerves. Among these, diabetic retinopathy (DR) is the leading cause of vision loss in working-age adults, arising from chronic hyperglycemia-induced damage to the retinal microvasculature and potentially progressing to blindness (2).

Histologically, DR presents as microangiopathy, characterized by vascular alterations such as focal capillary occlusion, venular dilation, arteriolar hyalinization, and thickening of the capillary basement membrane. Additional features, including pericyte loss and microaneurysm formation, reflect the progression of retinal injury (3).

Vision loss in DR is typically associated with the proliferative form (PDR) or diabetic macular edema (DME), both primarily driven by elevated vascular endothelial growth factor (VEGF) levels in response to retinal ischemia. VEGF-A promotes neovascularization and increases retinal vascular permeability (4). While VEGF-A is a key mediator of DR pathophysiology, other angiogenic pathways, such as those involving angiopoietin and angiotensinogen (AGT), also contribute significantly (5, 6). A deeper understanding of these additional pathways has led to the development of dual-action anti-angiogenic therapies, which aim to provide more effective and durable treatment outcomes (7).

Clinical studies suggest that certain antidiabetic medications may influence the progression and natural history of DR. For example, thiazolidinediones—an oral hypoglycemic class—have been associated with an increased risk of DME, likely due to their effects on fluid retention and vascular permeability (8). Moreover, anecdotal reports indicate a higher incidence of severe sight-threatening DR in patients receiving insulin therapy compared to those on oral agents, along with potential differences in DR outcomes among various insulin formulations (9, 10).

Synthetic insulins are produced by modifying a few amino acids of regular insulin to optimize pharmacokinetics; however, these modifications may also alter biological activity with possible clinical implications. Furthermore, insulin receptors have been identified in both neural and vascular retinal tissues, suggesting a direct role for insulin signaling in DR progression (11).

This study was designed to evaluate whether different insulin formulations exert differential effects on angiogenic gene expression in retinal cells. Specifically, it compares the effects of two rapid-acting insulins—glulisine and aspart—with those of standard regular insulin on VEGF-A and AGT gene expression in a hyperglycemic model using retinal pigment epithelial (RPE) cells and human retinal endothelial cells (HREC).

## Materials and methods

### RPE and HREC cell culture

Human eyeballs from an 18-year-old male organ donor were obtained from the local Eye Bank affiliated with Shiraz University of Medical Sciences and transferred to our laboratory under sterile conditions within 6 hours post-enucleation. Peripheral tissues were carefully removed from each eyeball. A small incision was made at the junction of the iris and sclera, allowing the intraocular contents to be gently evacuated using forceps. The eyeball was then opened further with scissors, and after thorough rinsing with sterile phosphate-buffered saline (PBS; Shellmax, Iran), the neural retina was carefully dissected for human retinal endothelial cell (HREC) culture. The remaining inner pigmented layer, corresponding to the retinal pigment epithelium (RPE), was completely separated from the inner surface of the eye using forceps.

For RPE cell culture, the pigmented inner layer—comprising the RPE/choroid complex—was sectioned into small fragments and cultured as explants in Dulbecco’s Modified Eagle Medium/Nutrient Mixture F-12 (DMEM/F12; Shellmax, Iran) supplemented with 10% fetal bovine serum (FBS; Gibco, Germany) and 1% penicillin/streptomycin (Shellmax, Iran) (12). Once the RPE cells reached confluence, they were trypsinized (passage 1) and further purified to eliminate any contaminating red blood cells. The purified cells were then expanded through successive passages to ensure cellular purity and consistency. To confirm the identity of the RPE cells, we assessed the expression of the RPE-specific marker RPE65 in both the first and sixth passages. PCR analysis using 100 ng of RNA followed by gel electrophoresis confirmed RPE65 expression in both passages, with reduced expression observed in passage six. This decline aligns with expected changes in RPE cells over successive cultures and supports the validity of their identity (12). These cells were subsequently used in downstream experiments, including insulin treatments and gene expression analysis by real-time PCR.

For the isolation of HRECs, we followed a protocol similar to that described by Zeng et al. (13). In brief, the neural retina, including the retinal blood vessels, was cut into 2 × 2 mm sections and incubated in 0.1% collagenase at 37°C for 30 minutes. The resulting cell suspension was filtered through a 70-μm cell strainer, centrifuged at 1500 rpm for 5 minutes, and cultured in DMEM/F12 medium supplemented with 10% FBS, 120 μg/ml penicillin, and 220 μg/ml streptomycin.

Notably, no coating agents were used for the culture of either RPE cells or HRECs. RPE and HREC cells between passages 6 and 8 were used for subsequent experiments. Although some native characteristics may be lost at these passages, they remain suitable for functional assays and gene expression analysis (12, 14).

### Study groups

To establish hyperglycemic, physiological, and osmolarity control conditions, cultured cells were exposed to the following environments for 24 hours. A culture medium containing 1 g/L glucose (equivalent to 5.5 mM) was used to simulate physiological conditions (physiological control group), while a medium containing 4.5 g/L glucose (equivalent to 25 mM) was used to induce hyperglycemia (hyperglycemic group). To match osmolarity without altering glucose concentration, a medium supplemented with 4.5 g/L mannitol (equivalent to 25 mM; D-Mannitol, Bio Basic, Markham, Canada) was used (osmolarity control group) (15, 16).

All culture conditions included serum-containing medium to better mimic the physiological environment. D-mannitol, a non-metabolizable sugar alcohol, was used in the osmolarity control group to replicate the osmotic pressure of the hyperglycemic medium without affecting cellular glucose metabolism. Previous studies have typically employed glucose concentrations ranging from 20 to 50 mM to model diabetic conditions in cell culture (17–19).

### Insulin treatment

**Figure 1:**
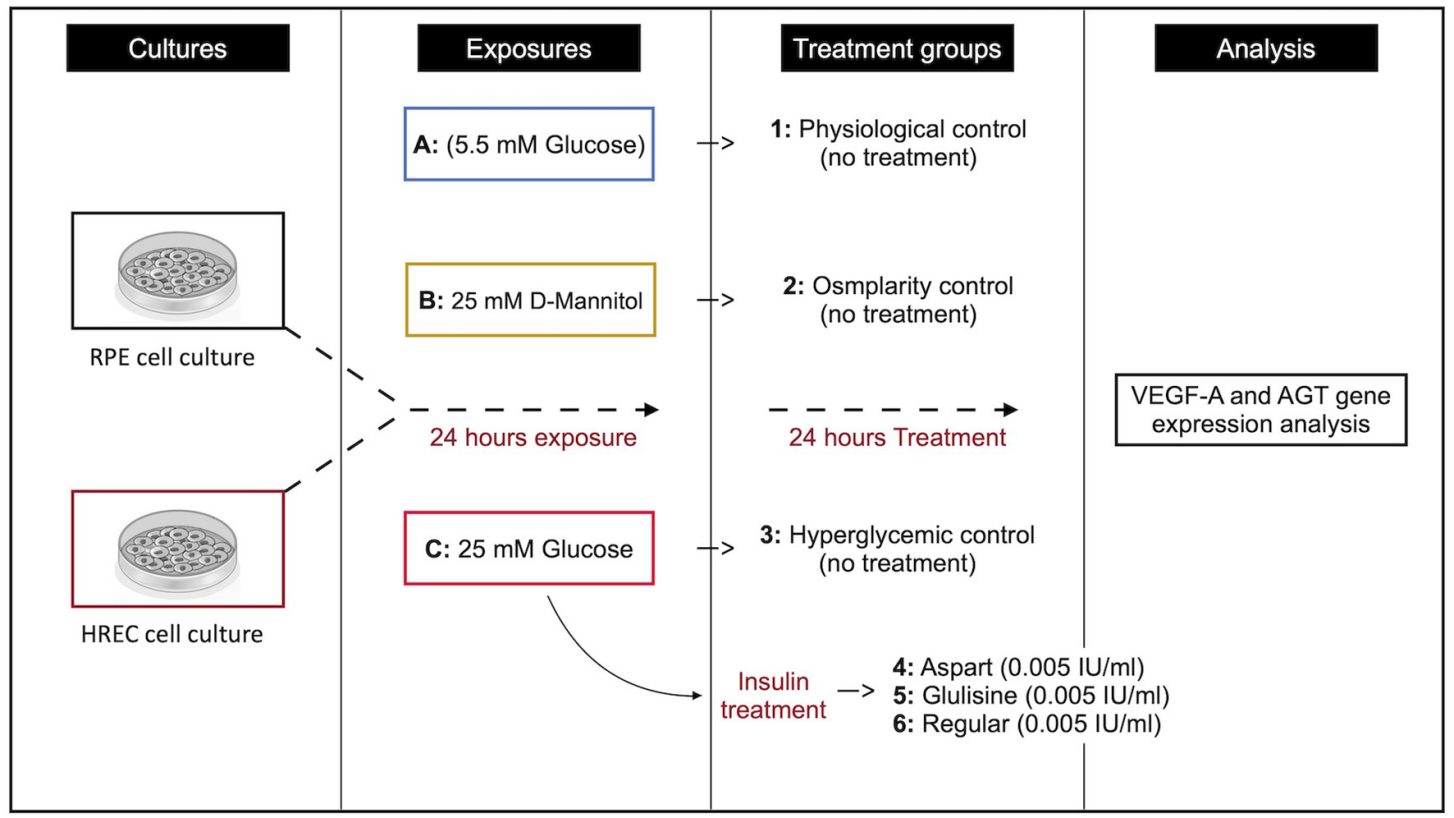
Schematic representation of the study design.

After 24 hours of exposure to the respective culture media, glulisine (Apidra^®^, Sanofi-Aventis, France), aspart (NovoRapid^®^, Novo Nordisk, Denmark), and regular insulin (LANSULIN^®^R, EXIR Pharmaceutical Co., Iran) were added to the media, and cells were incubated for an additional 24 hours. The experimental groups were as follows: Group 1 served as the physiological control (no drug treatment), Group 2 as the osmolarity control (no drug treatment), and Group 3 as the hyperglycemic control (no drug treatment). Group 4 received aspart insulin at a concentration of 0.005 IU/ml, Group 5 received glulisine insulin at the same concentration, and Group 6 received regular insulin at 0.005 IU/ml (see **Figure 1**). The insulin concentration used in this study (0.005 IU/ml) was derived based on previous reports, where a dosage of 0.2 IU/kg was administered to approximately 3000 ml of plasma (13). All treatments were conducted in serum-containing medium to better mimic physiological conditions and maintain cellular viability.

### Gene expression analysis

Primer sequences for the VEGF-A and AGT genes were designed using AllelID software and validated for specificity by BLAST analysis against the NCBI database. The specific primer sequences are listed in **Table 1**. Real-time PCR was performed to quantify the relative gene expression levels in treated versus control cells. Each 10 μL qPCR reaction consisted of 5 μL RealQ Plus 2× Master Mix Green (Ampliqon, Odense, Denmark), 1 μL of each forward and reverse primer (10 pmol/μL; Takapouzist, Iran), and 4 μL of diluted cDNA (1:10). The reactions were run on a Magnetic Induction Cycler (Mic qPCR, Bio Molecular Systems). PCR amplification followed a thermal cycling profile consisting of an initial denaturation at 95°C for 15 minutes, followed by denaturation at 95°C for 10 seconds, and annealing at 61°C for 45 seconds. Relative gene expression was calculated using the 2^-ΔΔCT^ method, with β-actin serving as the internal reference gene.

**Table 1:** The primer sequences of the genes under study.

Gene	Sense primer	Anti-sense primer	Product size
*VEGF-A*	ACGAACGTACTTGCAGATGTGAC	CGGCAGCGTGGTTTCTGTAT	137
*AGT*	CCACGCTCTCTGGACTTCAC	AGCCCTTCATCTTCCCTTGGA	166
* β-ACT*	GCCTCGCCTTTGCCGAT	CATGCCGGAGCCGTTGT	98 bp

All qPCR primers were custom synthesized by Takapouzist Company (Tehran, Iran) with HPLC purification. The primer sequences were designed using Primer-BLAST (NCBI) and validated for specificity via melt curve analysis.

### Statistical analysis

All experiments were conducted with *n* = 3 biological replicates per group, with each replicate representing an independent cell culture preparation. This sample size was determined based on power calculations from preliminary data and aligns with established standards for *in vitro* studies in comparable publications (12, 14). The *n* = 3 replicates were consistently applied across all experimental groups (Groups 1–6) and analytical methods (e.g., qPCR). The results are presented as mean ± standard deviation (SD). Statistical analyses were performed using SPSS software (version 26; SPSS Inc., Chicago, IL, USA). The normality of the data was assessed using the Kolmogorov-Smirnov test. A one-sample t-test was used to compare each treatment group to the standardized untreated control (set at 1). Depending on the experimental design, comparisons among treatment groups were conducted using either an independent t-test or one-way analysis of variance (ANOVA) followed by Tukey’s *post hoc* test. A P-value of <0.05 was considered statistically significant.

## Results

### Effect of different media on VEGF-A and AGT gene expression

**Figure 2:**
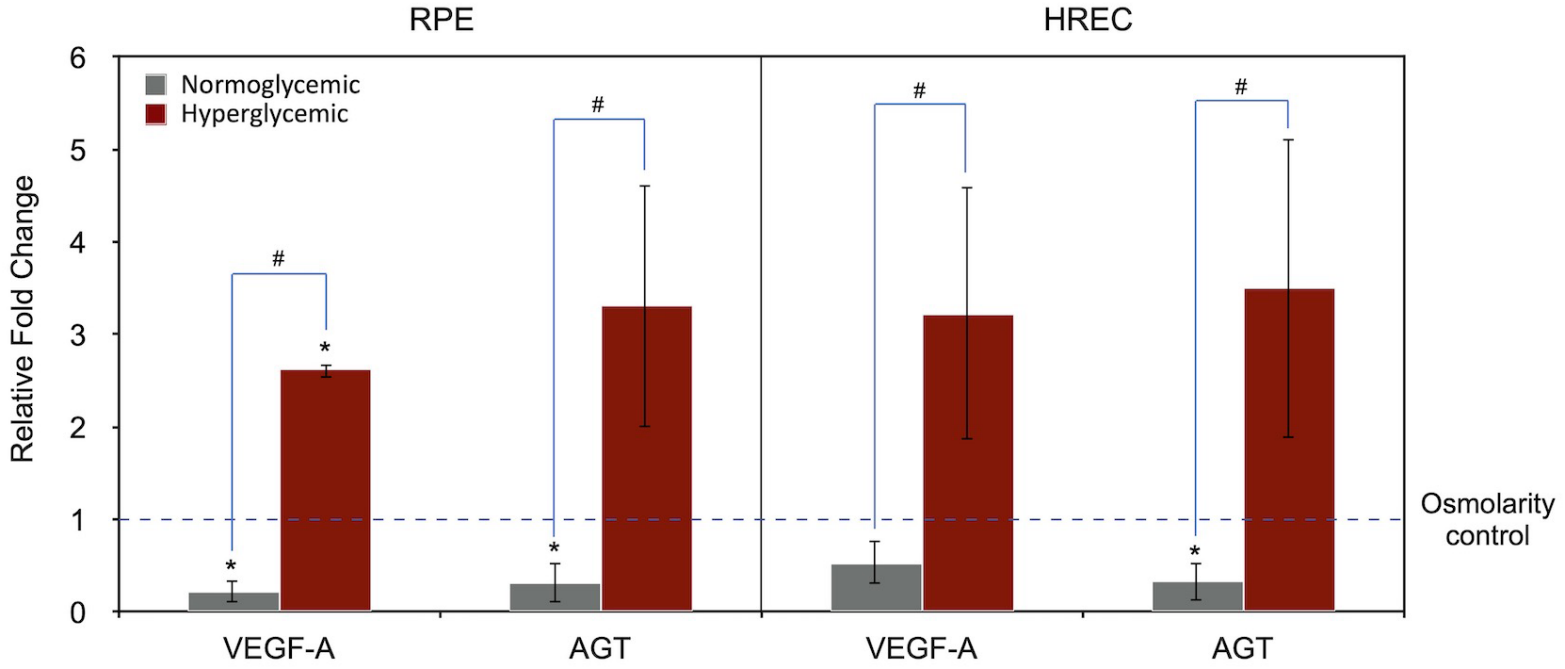
Expression of VEGF-A` and AGT genes under normoglycemic (physiologic; 5.5 mM, 1 g/L glucose) and hyperglycemic (25 mM, 4.5 g/L glucose) conditions in RPE and HREC cells, compared to the osmolarity control (25 mM D-mannitol; dotted line set at 1). Gene expression levels are normalized to the osmolarity control. Asterisks (*) above the error bars indicate p < 0.05 compared to the osmolarity control (one-sample t-test), while hashtags (#) above connecting lines indicate statistically significant differences between normoglycemic and hyperglycemic groups (independent t-test). RPE, retinal pigment epithelium; HREC, human retinal endothelial cells; VEGF-A, vascular endothelial growth factor-A; AGT, angiotensinogen.

**Figure 2** summarizes the effects of different media on VEGF-A and AGT gene expression in RPE cells and HRECs. In this analysis, data from both hyperglycemic and normoglycemic media were normalized to the osmolarity control medium (with osmolarity matched to that of the hyperglycemic medium), which was set to 1.

Compared to the osmolarity control, the hyperglycemic medium significantly increased VEGF-A expression in RPE cells (2.62-fold, P = 0.001, one-sample t-test) and showed a trend toward increased AGT expression (3.32-fold, P = 0.093). A similar pattern was observed in HRECs; however, the differences in VEGF-A (P = 0.107) and AGT (P = 0.116) expression did not reach statistical significance.

When comparing hyperglycemic with normoglycemic conditions, both VEGF-A and AGT expression were significantly higher in the hyperglycemic group across both cell types (RPE/VEGF-A, P < 0.001; RPE/AGT, P = 0.018; HREC/VEGF-A, P = 0.029; HREC/AGT, P = 0.028; independent t-test).

### Effect of different insulin formulations on VEGF-A gene expression

**Figure 3:**
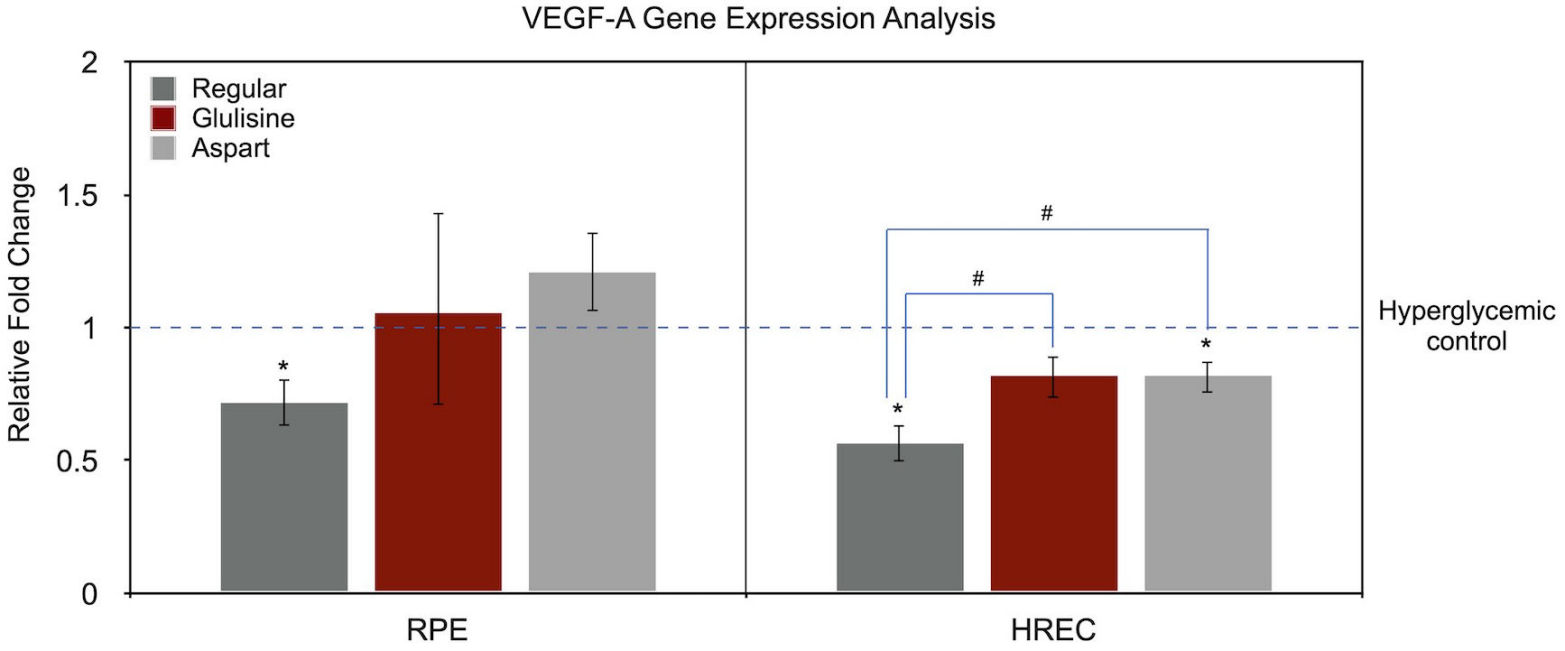
Effect of regular, glulisine, and aspart insulin on VEGF-A expression in HREC and RPE cells under hyperglycemic conditions. Gene expression levels are normalized to the untreated hyperglycemic control (dotted line set at 1). Asterisks (*) above the error bars indicate p < 0.05 compared to the untreated hyperglycemic control (one-sample t-test). Hashtags (#) above connecting lines denote statistically significant differences between insulin-treated groups, as determined by one-way ANOVA followed by Tukey’s *post hoc* test. RPE, retinal pigment epithelium; HREC, human retinal endothelial cells; VEGF-A, vascular endothelial growth factor-A.

**Figure 3** illustrates the effects of different insulin formulations on VEGF-A gene expression in hyperglycemic RPE cells and HRECs. The control condition was hyperglycemic medium without any treatment. Gene expression levels for each insulin treatment were normalized to the hyperglycemic control of the same cell type, which was set to 1.

In RPE cells, the only significant difference compared to the untreated control was observed with regular insulin (0.72-fold, *P* = 0.033, one-sample t-test). Aspart insulin showed a non-significant trend toward increased VEGF-A expression (1.21-fold, *P* = 0.136), while glulisine had no observable effect (*P* = 0.800). A one-way ANOVA yielded a *P* value of 0.096 when comparing all treatment groups, and the pairwise comparison between regular insulin and aspart showed a borderline difference (*P* = 0.090).

In the HREC cells, VEGF-A expression relative to the untreated hyperglycemic medium was as follows: regular insulin (0.57-fold, *P* = 0.009), glulisine (0.82-fold, *P* = 0.060), and aspart (0.82-fold, *P* = 0.035). ANOVA revealed a significant difference among treatment groups (*P* = 0.007). In pairwise comparisons, VEGF-A expression in regular insulin-treated cells was significantly different from both glulisine and aspart treatments (*P* = 0.011 for both), while no difference was found between aspart and glulisine (*P* = 1.00).

### Effect of different insulin formulations on AGT gene expression

**Figure 4:**
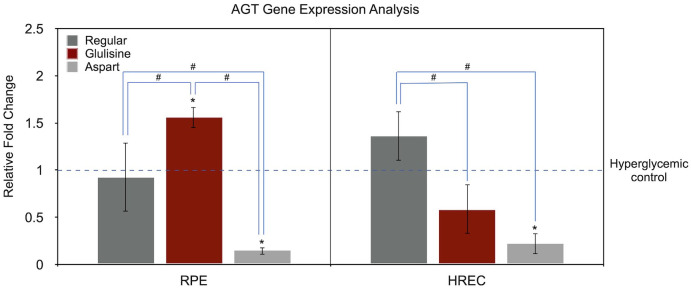
Effect of regular, glulisine, and aspart insulin on AGT expression in HREC and RPE cells under hyperglycemic conditions. Gene expression levels are normalized to the untreated hyperglycemic control (dotted line set at 1). Asterisks (*) above the error bars indicate p < 0.05 compared to the untreated hyperglycemic control (one-sample t-test). Hashtags (#) above connecting lines denote statistically significant differences between insulin-treated groups, as determined by one-way ANOVA followed by Tukey’s *post hoc* test. RPE, retinal pigment epithelium; HREC, human retinal endothelial cells; AGT, angiotensinogen.

**Figure 4** presents AGT gene expression analysis using the same approach as in **Figure 3**. In RPE cells, compared to the untreated hyperglycemic medium, glulisine significantly upregulated AGT expression (1.56-fold, *P* = 0.009, one-sample t-test), while aspart markedly downregulated it (0.15-fold, *P* < 0.001). Regular insulin had no significant effect (*P* = 0.680).

In HRECs, the only statistically significant difference was observed with aspart treatment (0.22-fold, *P* = 0.004). Regular insulin (1.36-fold, *P* = 0.097) and glulisine (0.58-fold, *P* = 0.074) showed marginal, non-significant trends.

One-way ANOVA revealed significant differences among insulin treatment groups in both RPE (*P* < 0.001) and HREC (*P* = 0.001) cells. In RPE cells, all three pairwise comparisons were statistically significant (regular vs. glulisine, *P* = 0.010; regular vs. aspart, *P* = 0.004; glulisine vs. aspart, *P* < 0.001). In HRECs, regular insulin differed significantly from both glulisine (*P* = 0.004) and aspart (*P* = 0.001), while the difference between glulisine and aspart did not reach statistical significance (*P* = 0.107).

## Discussion

In this study, it was demonstrated that hyperglycemic conditions significantly increased VEGF-A expression in both RPE and HREC cells compared to the physiologic normoglycemic condition (**Figure 2**). This finding is consistent with previous studies (20), which have identified VEGF-A as a key mediator in DR, primarily through its role in promoting angiogenesis and contributing to pathological neovascularization. Notably, the pattern of VEGF-A gene expression shown in **Figure 1** suggests that its upregulation under hyperglycemic conditions may be partially attributed to the hyperosmolar effect, when compared to physiological glucose levels, and partially due to other hyperglycemia-specific mechanisms.

We also observed a consistent upregulation of AGT gene expression under hyperglycemic conditions in both studied cell types. Although the role of AGT and the renin-angiotensin system (RAS) in DR progression is not yet fully elucidated, emerging evidence suggests that AGT upregulation may contribute to the transition toward the proliferative phase of DR (6, 27). The RAS pathway, which is traditionally recognized for its regulation of vascular homeostasis and fluid balance, has been implicated in inflammation and oxidative stress in diabetic tissues, further supporting its role in DR pathogenesis (21–23).

Experimental studies have confirmed AGT’s involvement in DR. In a rat model of DR, miRNA-133b promoted retinal vascular endothelial cell proliferation and inhibited apoptosis by targeting AGT through the AngII-ERK1/2 signaling pathway (24). Another study in the same model demonstrated dysregulation of the RAS pathway in microglia, suggesting that impaired microglial-vascular interactions may contribute to early vascular dysfunction in DR (25). Additionally, downregulation of AGT via overexpression of miRNA-29a prevented DR progression in another rat model (26).

In line with experimental studies, clinical reports further support the relevance of AGT in DR pathogenesis. A large proteomic analysis of human vitreous samples demonstrated significantly elevated AGT levels in patients with proliferative diabetic retinopathy (PDR) compared to those without DR (6). Additionally, local activation of the RAS has been reported in eyes with PDR, with Müller cells potentially contributing to this activation (27). Put simply, our findings reinforce previous reports on the role of AGT in the progression of DR by showing that hyperglycemic conditions increase AGT gene expression in two important human-retina derived cells: RPE and HREC. This may have clinical relevance, as AGT and the RAS could potentially be targeted as disease-modifying therapies.

In the management of diabetes, insulin therapy typically combines rapid-acting and long-acting insulin formulations to address both postprandial glucose spikes and basal insulin requirements. Rapid-acting insulin analogs, such as aspart, glulisine, and lispro, are developed through minor amino acid modifications of regular insulin, leading to altered pharmacokinetics that enable faster action while maintaining antiglycemic efficacy. However, these structural modifications may also influence alternative biological activities beyond glucose regulation (28–30).

Given the rich vascularization of the retina and choroid, along with the presence of insulin receptors in retinal cells (31, 32), systemic insulin therapy may have implications for DR progression. Indeed, some studies have suggested that insulin therapy may be associated with a greater risk of DR progression compared to oral hypoglycemic agents (10, 33). It was proposed that this effect may not be due to insulin itself, but rather to the rapid hypoglycemia it induces (10); however, on the other hand, certain insulin types such as lispro and glargine have been specifically linked to the progression of DR (9).

Our study provides preclinical evidence for the differential effects of various rapid-acting insulin analogues on VEGF-A and AGT—two key molecules implicated in the progression of DR—in two important retinal cells, HREC and RPE. As shown in **Figure 3**, regular insulin demonstrated a more favorable profile than either glulisine or aspart in downregulating VEGF-A expression in both cell types. While VEGF-A expression in HRECs showed a significant reduction with aspart and a similar but non-significant trend with glulisine, both analogues were less effective than regular insulin in this regard. This finding supports the notion that synthetic insulin analogues may possess altered biological properties beyond their intentionally modified pharmacokinetics.

In terms of AGT expression, aspart insulin demonstrated a more favorable profile, consistently reducing AGT levels in both RPE and HREC cells. In contrast, regular insulin had no significant effect, while glulisine exhibited opposing effects between the two cell types (**Figure 4**). These findings suggest that synthetic insulins—particularly aspart—may alter the progression of DR by mitigating hyperglycemia-induced upregulation of AGT and modulating the RAS pathway.

Notably, Insulin has also been shown to regulate the expression and activity of various components of the RAS in different cell types and tissues, including adipocytes and renal proximal tubular cells (34). Collectively, findings from the current and previous studies suggest that different insulin formulations may exert organ-specific protective or detrimental effects beyond their primary antiglycemic actions. Therefore, consideration of the health status of vulnerable organs—such as the eyes, kidneys, brain, and heart—may be important when selecting among available insulin therapies.

Finally, our study underscores the possible *interplay* between the RAS and VEGF-A pathways in hyperglycemic retinal injury. Previous research has demonstrated that angiotensin II can enhance VEGF-A expression, creating a feedback loop that exacerbates retinal pathology (35, 36). Importantly, a local RAS in the retina may independently contribute to DR progression (37, 38). Given the role of AGT in promoting VEGF-A expression, dual targeting of the RAS and VEGF-A pathways may offer a promising therapeutic strategy for DR (39).

### Powers and limitation

This study provides valuable insights into the effects of hyperglycemia on VEGF-A and AGT expression in two physiologically relevant retinal cell types and highlights the differential impacts of various insulin analogues. The use of simple and controlled experimental conditions enabled a focused analysis of gene expression under hyperglycemic stress. By investigating molecular-level mechanisms, this study adds important knowledge about the pathways involved in diabetic retinal complications and may help guide future therapeutic strategies.

However, several limitations should be acknowledged. Most notably, the *in vitro* design may not fully replicate the complex cellular and systemic environment of the diabetic human retina. The study primarily assessed short-term effects of insulin analogues, without evaluating their long-term impact—an important consideration in chronic conditions like DR. While hyperglycemia was the main focus, other key contributors such as inflammation and oxidative stress were not investigated, despite their established roles in disease progression. In addition, the analysis was limited to gene expression; due to budgetary and reagent constraints, protein-level validation could not be performed. Although we aimed to include all commonly used rapid-acting insulin formulations alongside regular insulin, lispro insulin was excluded due to its unavailability in our pharmacopeia. Finally, we acknowledge the importance of exploring additional genes (e.g., VEGF-C/D, placental growth factor, angiopoietin pathway, and insulin-like growth factor) and dose-dependent effects. While these experiments were beyond the scope and resources of the current study, they represent critical next steps for broader mechanistic insights. Future research using transcriptomic approaches (e.g., RNA-seq) and insulin concentration gradients will be essential for validating and extending our findings.

## Conclusions

In conclusion, this study demonstrated that hyperglycemia significantly upregulates VEGF-A and AGT expression in both RPE and HREC cells, underscoring a potential role for AGT in the pathophysiology of DR. The pattern of gene expression changes suggests that some effects of hyperglycemia may be attributed to hyperosmolarity, while others are likely due to glucose-specific mechanisms. Among the insulin types studied, regular insulin was most effective in downregulating VEGF-A expression in both cell types under hyperglycemic conditions. In contrast, aspart insulin consistently reduced AGT expression in both RPE and HREC cells. These findings may have important clinical implications, as different insulin formulations could exert distinct effects on the progression of DR—a possibility that warrants further investigation in clinical settings.

## Data Availability

The original contributions presented in the study are publicly available. This data can be found here: [Sanie-Jahromi, Fatemeh; Khosravi, Abtin; Hadianfard, Hooman; Nowroozzadeh, M. Hossein (2025), “Effects of insulin on vascular endothelial growth factor”, Mendeley Data, V1, doi: 10.17632/rtgb557t2x.1].
